# Neck circumference cutoff point as a predictor of metabolic syndrome in Brazilian rural workers

**DOI:** 10.1371/journal.pone.0316090

**Published:** 2025-01-07

**Authors:** Manoela Cassa Libardi, Cleodice Alves Martins, Júlia Rabelo Santos Ferreira, Glenda Blaser Petarli, Monica Cattafesta, Olívia Maria de Paula Alves Bezerra, Eliana Zandonade, Carlos Eduardo Gomes Siqueira, Luiz Carlos de Abreu, Jonathan Filippon, Luciane Bresciani Salaroli

**Affiliations:** 1 Graduate Program in Collective Health, Health Sciences Center, Universidade Federal do Espírito Santo, Vitória, Brazil; 2 Health Sciences Center, at the Universidade Federal do Espírito Santo, Vitória, Brazil; 3 Universidade Federal de Ouro Preto, Ouro Preto, Brazil; 4 Massachusetts University, Boston, Massachusetts, United States of America; 5 Queen Mary University of London, London, United Kingdom; 6 Graduate Program Nutrition and Health, Health Sciences Center, Universidade Federal do Espírito Santo, Vitória, Brazil; Endocrinology and Metabolism Population Sciences Institute, Tehran University of Medical Sciences, ISLAMIC REPUBLIC OF IRAN

## Abstract

Neck circumference (NC) is a predictive measure for the diagnosis of Metabolic Syndrome (MS). The aim of the present study was to establish cutoff points for NC as a predictor of the presence of MS in Brazilian rural workers, based on the MS components according to the IDF and NCEP-ATP III criteria. This is a cross-sectional study carried out with rural workers in the municipality of Santa Maria de Jetibá, in the state of Espírito Santo, Brazil. The ROC curve was calculated and the cutoff points for predicting the risk of developing MS were stipulated from the NC, identified by the area under the curve, using different methods of criteria for determining MS. Sensitivity, specificity, positive and negative predictive values and Youden index were applied. The significance level adopted was 5%. The cutoff points were different for males, resulting in 39.550 cm (AUC 0.832) according to the NCEP-ATP III criterion and 39.125 cm (AUC 0.888) according to the IDF criterion. For women, the cutoffs were similar, resulting in a single cutoff of 34.725 cm (AUC 0.862 for NCEP-ATP III and 0.849 for IDF). The cutoff points defined for men and women for NC showed good sensitivity and specificity for predicting MS in the studied population. The NC measurement proved to be a simple, low-cost and accurate measure for assessing this morbidity in Brazilian rural workers.

## Introduction

Metabolic syndrome (MS) corresponds to the set of metabolic changes that, when present in the individual, increase the chance of developing cardiometabolic diseases, such as heart disease, stroke and diabetes. These factors are related to insulin resistance and increased blood glucose, blood pressure, dyslipidemia, obesity and central body fat deposition [1–3]. MS is still considered a possible positive predictor of morbidity and mortality from cardiovascular diseases [4].

Anthropometric assessment is one of the criteria for the diagnosis of MS and is commonly used to measure body parameters as it is a method used internationally and applied to assess indicators in large populations [5, 6] due to it being low cost, easy to apply and noninvasive [7, 8].

Neck circumference (NC) is an anthropometric measurement recommended for use in clinical practice as a low-cost and easy-to-train method [9] for the assessment of excess body weight, identified as a marker of the distribution of subcutaneous adipose tissue in the upper body, contributing to the diagnosis of overweight, obesity and associated diseases [10], and can be recommended for use in Primary Health Care, as it is a cheap and easy training method [9].

The main advantages of using neck circumference in relation to other measurements are the simplicity of the measurement method, in addition to being quick and non-invasive, not undergoing changes during the day, not being influenced by issues related to fasting and satiety and the prandial period, have very low cost of measuring instruments. Other important factors are the possibility of use in patients with reduced mobility, such as bedridden and hospitalized patients, and the non-need to be wearing little clothing at the time of the assessment, not exposing the patient who, for any reason, psychological, cultural or religious, may feel embarrassed during an assessment [9, 10].

Despite its importance, there are few studies that relate the use of NC cutoff points to predict MS and other chronic diseases in rural workers [11–14], and so far there is no consensus on the definition of cutoff points and studies published in Brazil for this population, this being an unprecedented study.

This study aims to establish cutoff points for NC as a predictor of the risk of developing MS in Brazilian rural workers, based on MS components according to the International Diabetes Federation (IDF) and the National Cholesterol Education Program’s Adult Treatment Panel III (NCEP-ATP III).

## Methods

### Study design and population

This is a cross-sectional epidemiological study, part of the project “Health condition and associated factors: a study with rural workers in Espírito Santo—AgroSaúdES,” funded by the Fundação de Amparo à Pesquisa e Inovação do Espírito Santo (FAPES).

This study has been approved by the Research Ethics Committee of the Health Sciences Center of the Federal University of Espírito Santo, reference number 2091172 (CAAE 52839116.3.0000.5060), meeting the requirements demanded by the Resolution of the National Health Council nº 466/ 12 and the Declaration of Helsinki and its supplements for research involving human beings. Subjects signed the written Free and Informed Consent Term to participate in the study.

The study was carried out in the municipality of Santa Maria de Jetibá, in the state of Espírito Santo, located in southeastern Brazil, and obtained a representative sample of rural workers, according to the following inclusion criteria: age between 19 and 59 years; not being pregnant; having agriculture as the main source of income; and being at least six months in full employment.

To identify eligible rural workers in the original study, data available in the individual and family records of the Family Health Strategy teams were used, covering 100% of the 11 health regions of the municipality, minimizing potential selection biases related to unequal access to health services. The survey identified 4,018 families and a total of 7,287 rural workers. From this universe, a sample was calculated for the original project considering 50% of expected prevalence of abdominal obesity (to maximize the sample), 3.5% of sampling error and a significance level of 95%, making a minimum sample of 708 rural workers for the original study. A total of 806 rural workers were invited to compensate for possible losses. All sample size calculations were performed using the EPIDAT program (version 3.1).

The study participants were selected through a stratified random sampling process, taking into account the number of families in each health region as well as the number of Community Health Agents (CHAs) in the area, ensuring proportional representation across the 11 regions and the 80 CHAs. To prevent duplication of information, only one individual per family was chosen. In the event of refusal or non-attendance, another individual from the reserve list was chosen. In the event of refusal or non-attendance, another individual from the same gender classification and health unit as the original participant.

Data collection took place from December, 8, 2016 to April, 4, 2017 on the premises of the municipality’s health units by trained researchers. The details involved in data collection and research development are described in the article by Petarli and collaborators [15].

### Data collection

Blood was collected from rural workers for biochemical tests. To minimize errors, the analyzes were carried out by a single laboratory and a 12-hour fasting period was determined for carrying out the tests.

Total cholesterol, HDL-c, LDL-c and triglyceride levels were measured. Total cholesterol and HDL cholesterol were determined, respectively, by the enzymatic colorimetric method with the Liquicolor Cholesterol Kit (In Vitro Diagnostica Ltda) and the HDL Cholesterol Precipitation Kit (In Vitro Diagnostica Ltda). Triglycerides were determined by the enzymatic colorimetric method with the Liquicolor mono^®^ Triglycerides Kit (In Vitro Diagnostica Ltda). Individuals who reported using lipid-lowering medications were also considered to have dyslipidemia. The more detailed analysis of lipid profile collection is described in the article by Petarli et al. [15].

The classification of blood pressure levels in the study was based on the criteria defined by the VII Brazilian Guidelines on Hypertension [16], considering as hypertensive individuals with systolic blood pressure (SBP) equal to or greater than 140 mmHg and/or diastolic blood pressure (DBP) equal to or greater than 90 mmHg, or those who were taking medications to blood pressure control. Measurements were taken during the interview, using the Omron^®^ HME-7200 Automatic Pressure Monitor, calibrated by the National Institute of Metrology, Quality and Technology (INMETRO), and each participant had their pressure measured at least three times. To ensure the accuracy of the results, participants rested for around five minutes before the assessment, avoided food, alcoholic beverages, coffee or cigarettes in the 30 minutes before the exam, and emptied their bladder beforehand. The analysis considered the average of the first two measurements, with a third measurement being carried out when the difference between the first two exceeded 4 mmHg [15]. The more detailed analysis of lipid profile collection is described in the article by Petarli et al, [15].

The collection of anthropometric measurements was detailed in the article by Prado et al. [17]. Weight was measured with participants barefoot, in an upright position, wearing as little clothing as possible using a Tanita^®^ portable scale. Height was measured with individuals barefoot, standing upright, arms extended along the body and eyes fixed on a point on the horizon with a Sanny^®^ portable stadiometer. Waist circumference (WC) was measured with the participant standing, arms extended along the body and feet together, with the inelastic tape at the midpoint between the lower edge of the costal arch and the iliac crest. Three non-consecutive measurements were taken, the first being discarded and the average of the last two considered as the final measurement. The WHO [18] cutoff points were used to classify BMI into eutrophic/underweight and overweight/obese.

The NC was obtained with the individual with the head in the Frankfurt position, that is, with the eyes facing forward, placing a flexible inextensible measuring tape Sanny model TR-4010^®^ (Promohealth trade of medical and specialized products, Bauru, São Paulo, Brazil), at the point just below the prominence of the larynx and was expressed in centimeters [19].

For all measurements, three non consecutive repetitions were performed, the first being discarded and the average of the last two considered as the final measurement. The equipment was calibrated and validated by the National Institute of Metrology, Quality and Technology (INMETRO).

To establish the NC cutoff point for both genders, the MS criteria according to the IDF and NCEP-ATP III were used. For the IDF, individuals are considered to have MS if they have abdominal obesity assessed by a WC ≥ 84 cm for women and ≥ 94 cm for men, with the presence of two more criteria: fasting glucose ≥ 100 mg/dL; SBP ≥ 130 mmHg or DBP ≥ 85 mmHg; TG ≥ 150 mg/dL and HDL-c < 40 mg/dL for men and < 50 mg/dL for women. Regarding the NCEP-ATP III, individuals were considered to have MS with the following criteria: WC > 88 cm for women and > 102 cm for men; HDL-c < 40 mg/dL for men and < 50 mg/dL for women; TG ≥ 150 mg/dL; SBP ≥ 130 mmHg or DBP ≥ 85 mmHg and fasting blood glucose ≥ 100 mg/dL. For both classifications, the use of antihypertensive, hypoglycemic and/or medication for dyslipidemia were considered criteria for MS, as they classify the individuals with hypertension, diabetes and/or dyslipidemia, respectively [1].

### Statistical analysis

To describe the study variables, absolute and relative frequencies were used. Data were subjected to receiver operating characteristic (ROC) curve analysis to establish the cutoff points for NC, according to the set of conditions that make up the criteria for MS in both diagnostic criteria mentioned above, using the 95% confidence interval. ROC analysis is a tool to evaluate the diagnostic accuracy of a test and determine the best cutoff point considering sensitivity and specificity. Cutoff points were defined based on the Youden Index, specificity and sensitivity.

Although the ROC curve is often associated with logistic models, in the present study, the analysis was used as an independent method to determine the ideal cutoff point for the evaluated neck circumference, not depending on predictive modeling. We also emphasize that the configuration of the function used in the analysis is equivalent to a logistical approach, as discussed in the literature [20].

Given the objective of the analysis, no confounding factors were considered in the analysis to investigate associations between multiple variables or causal relationships, which focused on the performance of the diagnostic test, on identifying an ideal cutoff point.

All analyses were performed using the R software (4.0.3) for Windows. The significance level adopted was 5%.

## Results

In total, 790 rural workers were evaluated. There were no data losses from the 790 rural workers who agreed to participate in the research, all of their data was collected, of which 413 (52.3%) were men and 377 (47.7%) women. Regarding age group, 213 (27%) were 30 years old or younger, 231 (29.2%) between 31 and 40 years old, 195 (24.7%) between 41 and 50 years old and 151 (19.1%) over 50 years old. In the sample, there was a predominance of white individuals (702, 88.9%) and individuals with up to 4 years of schooling (533, 67.5%), followed by studies between 5 and 8 years (173, 21.9%) and over 8 years of study (84, 10.6%).

The predominant economic class found in the sample was class C, with 395 participants (50%), followed by class D/E with 337 participants (42.7%) and class A with 58 participants (7.3%). In relation to hours worked weekly, 628 (79.5%) worked 40 or more hours per week and 609 (77.1%) owned the land where they worked.

The prevalence of each component of metabolic syndrome for both criteria and genders is shown in Table 1.

**Table 1:** Prevalence of each component of metabolic syndrome according to IDF and NCEP-ATP III.

**IDF**	**Men n (%)**	**Women n (%)**
Abdominal obesity (WC ≥ 84 cm for women and ≥ 94 cm for men)	138 (17.5)	226 (28.6)
Glucose ≥ 100 mg/dL	19 (2.4)	14 (1.8)
SBP ≥ 130 mmHg or DBP ≥ 85 mmHg	243 (30.8)	118 (14.9)
TG ≥ 150 mg/dL	74 (9.4)	51 (6.5)
HDL-c < 40 mg/dL for men and < 50 mg/dL for women	46 (5.8)	96 (12.2)
**NCEP-ATP III**	**Men n (%)**	**Women n (%)**
Abdominal obesity (WC > 88 cm for women and > 102 cm for men)	61 (7.7)	184 (23.3)
HDL-c < 40 mg/dL for men and < 50 mg/dL for women	46 (5.8)	96 (12.2)
SBP ≥ 130 mmHg or DBP ≥ 85 mmHg	243 (30.8)	118 (14.9)
TG ≥ 150 mg/dL	74 (9.4)	51 (6.5)
Glucose ≥ 100 mg/dL	19 (2.4)	14 (1.8)

For both criteria, high blood pressure was the most common component of MS for men (30.8% for IDF and NCEP-ATP III), while abdominal obesity was the most common component for women (29.6% for IDF and 23.3% for NCEP-ATP III). The lowest prevalence was of high blood glucose for both genders, assuming the same percentages for both criteria (2.4% for men and 1.8% for women).

**Fig 1:**
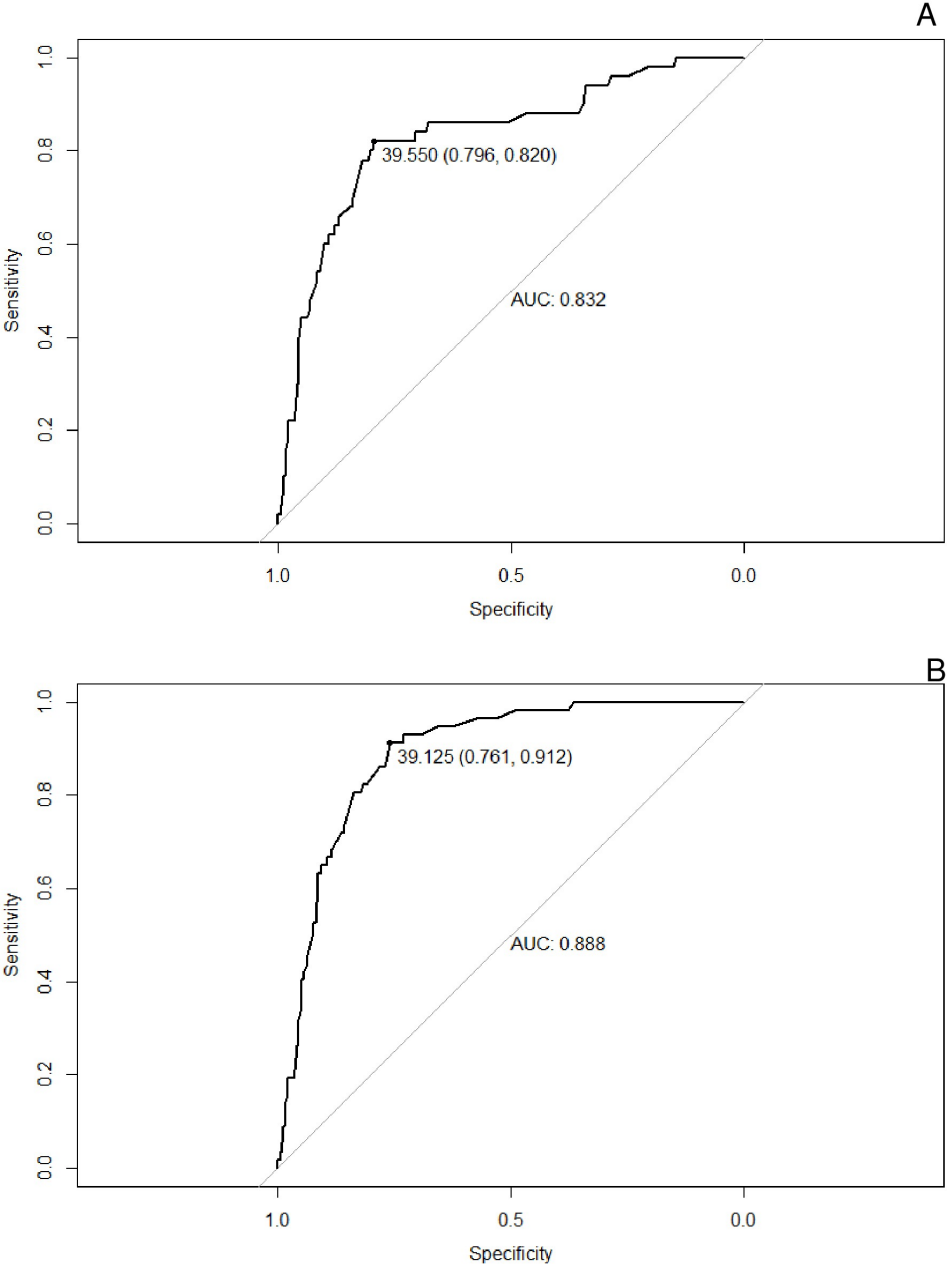
NC ROC curve for the diagnosis of MS in men, using the NCEP-ATP III (1A) e IDF (1B) criteria, respectively. AUC: area under the curve; MS: metabolic syndrome; NC: neck circumference; NCEP-ATP III: National Cholesterol Education Program; IDF: International Diabetes Federation.

The analysis of the ROC curve for males is shown in Fig 1. For the NCEP-ATP III criterion, the area under the curve (AUC) was 0.832 (CI = 0.766–0.897, p < 0.001) and for the IDF criterion, the area under the curve (AUC) was 0.888 (CI = 0.850–0.926, p < 0.001).

**Fig 2:**
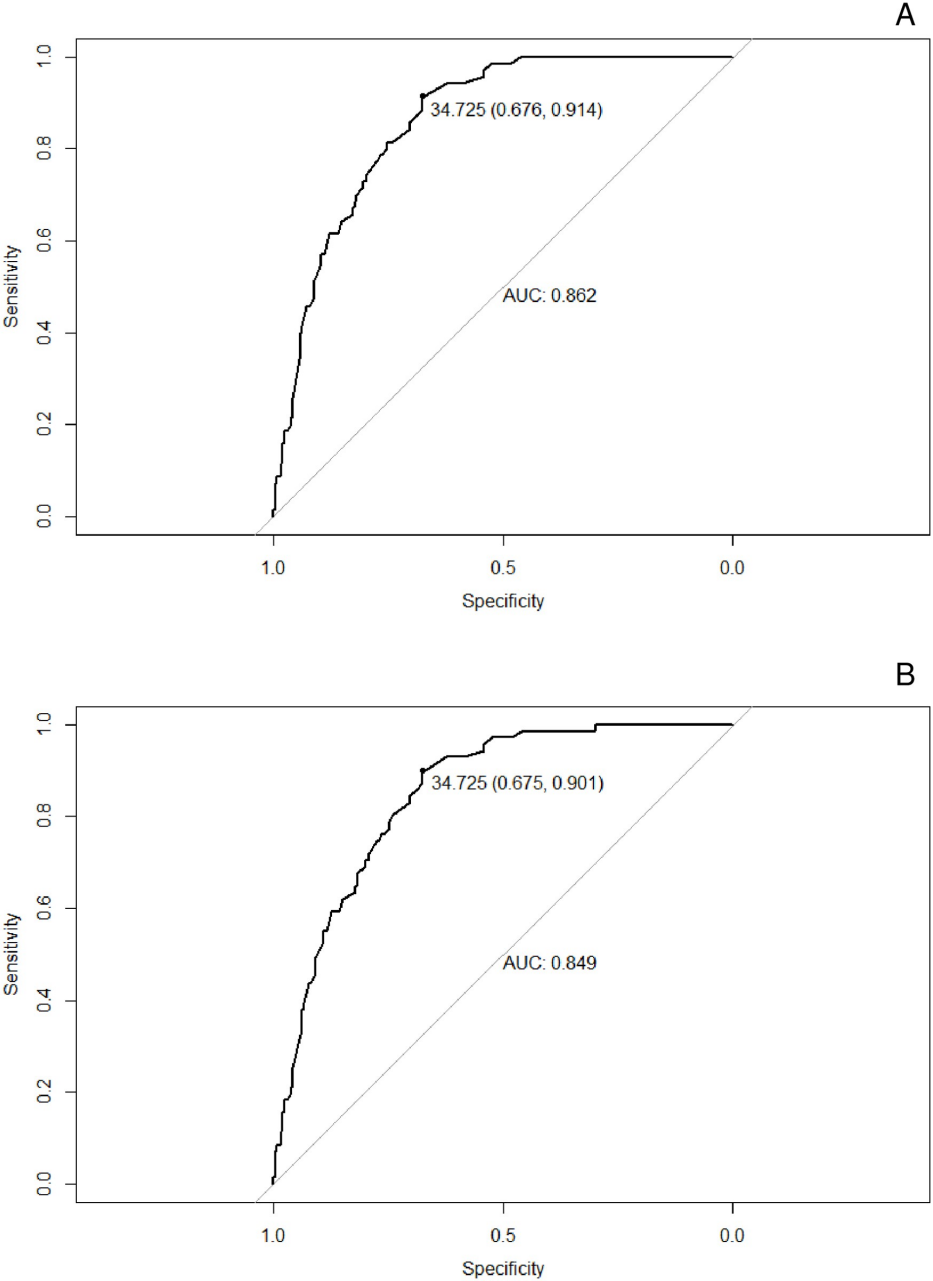
NC ROC curve for the diagnosis of MS in women, using the NCEP-ATP III (2A) e IDF (2B) criteria, respectively. AUC: area under the curve; MS: metabolic syndrome; NC: neck circumference; NCEP-ATP III: National Cholesterol Education Program; IDF: International Diabetes Federation.

The results of the analysis of the ROC curve for females are shown in Fig 2. For the NCEP criterion, the AUC was 0.862 (CI = 0.823–0.902, p < 0.001) and for the IDF criterion, the AUC was 0.849 (CI = 0.807–0.892, p < 0.001).

For males, the cutoff points were different between the NCEP and IDF criteria, namely 39.550 (Youden Index 0.616) and 39.125 (Youden Index 0.673), respectively. For women, the cutoff points were the same between the NCEP-ATP III and IDF criteria, 34.725 (Youden Index 0.590 and 0.576, respectively), as shown in Table 2.

**Table 2:** Cutoff points and diagnostic performance measures for NC by gender for the diagnosis of MS.

Variables	Men 95% CI		Women 95% CI	
	NCEP-ATP III	IDF	NCEP-ATP III	IDF
Cutoff points	39.550	39.125	34.725	34.725
AUC	0.832 (0.766–0.897)	0.888 (0.850–0.926)	0.862 (0.823–0.902)	0.849 (0.807–0.892)
Accuracy	0.799 (0.798–0.799)	0.782 (0.781–0.782)	0.721 (0.720–0.722)	0.718 (0.717–0.719)
Sensitivity	0.820 (0.714–0.926)	0.912 (0.839–0.986)	0.914 (0.849–0.980)	0.901 (0.832–0.971)
Specificity	0.796 (0.754–0.837)	0.761 (0.716–0.805)	0.676 (0.624–0.729)	0.675 (0.623–0.728)
PPV	0.357 (0.269–0.444)	0.380 (0.298–0.461)	0.393 (0.318–0.468)	0.393 (0.318–0.468)
NPV	0.970 (0.950–0.989)	0.982 (0.966–0.998)	0.972 (0.950–0.994)	0.967 (0.943–0.991)
Youden Index	0.616	0.673	0.590	0.576

AUC: area under the curve; PPV: positive predictive value; NPV: negative predictive value; CI: confidence interval.

## Discussion

This is the first study to identify NC cutoff points for the diagnosis of MS in rural workers in Brazil. This measurement is a known method of anthropometric assessment; however, it is still seldomly used in clinical practice, due to the absence of specific standardized cutoff points as a reference for the diagnosis of diseases and comorbidities. In rural populations, studies are even more scarce.

Measurements considered the gold standard for assessing body fat, such as ultrasound, magnetic resonance imaging and computed tomography, are not part of a clinical routine in health services, in addition to being high-cost [21]. In this sense, anthropometric measurements are indicated to be used on a large scale, meeting the need to work on the early identification of risk factors for cardiometabolic risk in Primary Health Care, with instruments that allow professionals and patients to feel comfortable with the assessment.

Thus, neck circumference becomes a simple, accessible and easy-to-measure alternative, with great acceptance among health professionals and patients, and can be used to screen patients in practice and in an effective and practical way in routine medical environments. clinical trials for large populations [5, 8, 22].

Among the main advantages of using CP measurement as a screening method, we can mention: it is a quick and non-invasive, low-cost method, which does not change during the day, regardless of the moment of measurement, pre or postprandial [9, 21], as well as not being affected by skeletal deformities, intra-abdominal conditions caused by pathologies or amputations [5], as well as in hospitalized patients and with reduced mobility [9]. It is not affected by temperature or sociocultural limitations. It has very low instrument costs, training for measurement is easy; Furthermore, it can be used in patients with reduced mobility, such as bedridden and hospitalized patients. Importantly, it does not expose the patient, for any reason, psychological, cultural, religious or embarrassment, which other traditional methods require, such as wearing few clothes at the time of the assessment [9, 10, 21] and is also independent of the season, as it does not require the removal of clothing for measurement [8].

The impact of chronic diseases on the lives of rural workers, on productivity and on healthcare costs needs to be considered, as factors such as difficulty in accessing health services and specialized care further increase the vulnerability of this population, contributing to the development of these conditions [15].

Data on the prevalence of each component of MS indicate that regardless of the criteria used, IDF or NCEP-ATP III, abdominal obesity and hypertension were the most prevalent conditions. In men, hypertension was particularly more prevalent according to both definitions and in women, abdominal obesity had a higher prevalence. On the opposite, high glucose was the least common criterion in both genders and in both definitions.

In the study by Petarli (2019) [15], carried out with the same population as the present study, 80% of rural workers had at least one chronic disease, more than 40% had two or more and 17% had three or more chronic diseases, with predominance of endocrine, nutritional or metabolic diseases.

Another study carried out with the same population found that the main risk factors that increased the risk of developing MS were gender (women presented a higher risk), age (in the range between 41 and 50 years and over 50 years), BMI (overweight individuals at greater risk than eutrophic or underweight individuals), and land ownership (not owning it was a risk factor) [23].

The same authors also found that low HDL, increased blood pressure and high waist circumference were the most prevalent components of MS. Among the five variables used in the diagnosis of MS, there is a great difference in the proportion of high waist circumference for both criteria (IDF and NCEP-ATP III), with a markedly higher prevalence in women [18]. Abdominal obesity, assessed by waist circumference, is one of the fundamental components of MS, also reflecting the increased risk of cardiovascular disease [6]. Thus, the use of easy-to-use nutritional diagnostic indicators is important to assess the distribution of body fat and identify obesity early [24].

Although WC is the most used measurement, NC is a better predictor of central and higher obesity [25]. This occurs because the accumulation of visceral fat and fat in the upper part of the body is associated with the increased release of free fatty acids into the circulation, contributing to increased insulin resistance and vascular dysfunction, and consequently increased cardiometabolic risk. Systemic circulation provides most of the free fatty acids that reach the liver [25–27]. The greater the accumulation of fat in the upper part of the body, the greater evidence of abnormal metabolism functioning can be found, such as the development of diabetes, insulin resistance, hypertension and dyslipidemia [25–27]. Furthermore, the accumulation of body fat associated with a chronic inflammatory state is an important contributor to insulin resistance and central obesity is an essential component of the metabolic syndrome [9].

The results found in the present study show that differences were found in the cutoff points defined for the male population, contrary to the results observed in the female population, which presented the same value for both criteria. This fact can be explained by the lower variability of the data collected in the female population, compared to the male population, in relation to the criteria used for the diagnosis of MS.

Vague (1956) [28] proposed for the first time the association between the accumulation of fat predominantly in the upper part of the body with metabolic disturbances; however, for several decades, studies were focused on the evaluation of body fat distribution from WC measurements and hip circumference (HC) and the waist–hip ratio (WHR) [29].

Currently, studies point out that the location of body fat is an important factor in determining health risk and the development of cardiometabolic diseases [30], since the deposition of the upper body, such as the neck, seems to be related to a greater release of acids plasma free fatty acids, with a greater relation to increased cardiovascular risk and greater insulin resistance, such as visceral fat [31, 32].

Thus, interest in studies of the association between body fat distribution in the upper body has increased, specifically in research regarding the neck region [33], recognizing its relationship with cardiometabolic risk, independent of other measures of adiposity [32, 34].

Studies over the last decade have shown that the NC measurement has a strong association with WC, HC, WHR and BMI measurements and the identification of obesity [5, 21, 25, 35–37].

It was also evaluated that NC is an important predictor for the diagnosis of overweight and obesity [38], MS [25, 39] and central obesity [25], in addition to being associated with glucose intolerance, hyperinsulinemia, diabetes and hypertriglyceridemia [21] and being an important anthropometric measure to assess cardiovascular risk factors [25, 40], increased blood pressure, LDL-c [8, 35], inflammatory markers [41] and coronary artery disease [42], predicting a higher incidence of fatal and nonfatal cardiovascular events [40].

Ebrahimi et al. [25], in 2021, evaluated the ideal NC cutoff point for the diagnosis of MS in the adult Iranian population and observed that the measure was a predictive factor for the development of MS, showing correlation with central obesity, increased fasting glucose, arterial hypertension and dyslipidemia. In this population, the NC defined for the diagnosis of MS was 36 cm for women and 42 cm for men, values above those found in the present study.

In contrast, lower cutoff points were determined in a survey carried out with a Korean population, aged 40 to 65 years. NC was positively correlated with BMI and WC in men and women, increased blood pressure and dyslipidemia. According to the authors, the cutoff points of 38.5 cm for men and 33.65 cm for women were able to predict the development of MS in this population [35].

Hoebel, Malan and Rider (2012) [43] evaluated male and female adult African and Caucasian teachers in South Africa. NC determined the risk for MS in all Caucasian groups. The groups of Caucasian men and African women had cutoff values above those found in the present study, with measurements of 40 and 41 cm for younger and older Caucasian men, respectively, and 35 cm for older African women. It is currently known that age contributes to the onset of MS and other metabolic disorders [44].

Similar associations were found in a recent study by Zanuncio et al. in 2022 [39], where increased CP was associated with greater risks of developing MS. The authors found values of 39.5 cm and 33.3 cm for men and women, respectively.

In the study by Silva et al. (2020) [32], it was observed that a 1 cm increase in NC was associated with a 3% increase in the arithmetic mean risk of cardiovascular disease in men and 5% in women. The authors also observed a positive association between the increase in NC and the cardiovascular risk in 10 years, suggesting the findings of the study to estimate the cardiovascular risk.

However, none of the cited studies that found associations between NC and MS were carried out with rural populations of the countries.

Among the few studies that use the diagnosis of MS based on the identification of NC cutoff points for the rural population, we found the following: in Ukraine, NC cutoff points of 36.5 cm for women and 38.5 cm for men were defined for the diagnosis of type 2 diabetes mellitus [11]; in Thailand, NC was associated with systemic arterial hypertension above 37.5 cm for men and 32.5 cm for women [12]; In India, in both urban and rural populations, high and normal NC was positively correlated with BMI and increased blood pressure, with greater statistical significance in the urban population when compared to the rural population [45].

Obesity and related diseases, such as MS, can be related to food insecurity and malnutrition, critical determinants of health.

Malnutrition is the main cause of illness in the world [46]. The worsening of living and working conditions, topped up and increased by social inequality, can affect basic living conditions and needs, such as access to food [47]; the effects on the acquisition of food can be the reduction or total absence of fruits and vegetables purchases, while likely increasing the consumption of ultra-processed and ready-to-eat foods [48].

This situation leads the population to a greater risk of developing non transmissible chronic diseases related to food, such as obesity, high blood pressure, diabetes and MS, due to a diet of low nutritional quality [49, 50]. Decreased consumption of culinary ingredients can increase the risk of abdominal fat accumulation by between 1.57 and 1.66 times [17].

In the study by Cattafesta et al. (2020), carried out with the same population as the present study and previously published, the higher caloric intake of ultra-processed foods was associated with the higher caloric content of the diet and the lower consumption of all 23 nutrients analyzed [51].

In Brazil, food insecurity is greater in rural regions of the country and is present in more than 60% of households, including those where there are rural workers and small farm producers [52].

Thus, the results found could be used as an anthropometric marker for early and periodic assessment of body fat distribution, to identify the risks associated with MS in clinical practice and in epidemiological studies for the studied population, due to its association with central adiposity. and cardiometabolic risk.

Due to the need to use only one measuring tape to perform the measurement, which further highlights the practicality and cost-benefit of the measurement, the incorporation of CP as a measurement can be easily carried out in the health system, especially in the scope of primary care, regions of social vulnerability, with basic infrastructure, in rural or difficult-to-access areas, with limited resources or difficulty in acquiring advanced diagnostic technologies and laboratory tests.

This is an effective and low-cost way to increase prevention and early action by healthcare professionals based in PC facilities in rural areas; Due to its ease of application and replication among health workers, including by community health agents themselves, it could be implemented as an initial screening and part of routine anthropometric reviews, whether in PHC, in routine consultations and individual or collective approaches or in home visits, which are common practices in the Brazilian Unified Health System, especially in communities that may have difficulties in accessing services, where primary care often becomes the only access door to health care, as in the population rural.

Such preventive actions are especially relevant in a scenario of cost containment in the medium and long term of health financing in low and middle income countries such as Brazil, as they offer numerous positive factors, allowing preventive measures against NCDs and probably reducing the use of resources related to future health care utilization.

The limitation of this study is its cross-sectional nature, which must be taken into account when interpreting the results found due to the possibility of reverse causality, considering that the evaluation was carried out at a single moment, not establishing a cause and effect relationship between the variables nor changes over time. Therefore, even though the data are representative, it is not possible to affirm the effect of one variable on the other, which requires caution when interpreting the associations observed between the variables. Furthermore, due to the availability of few articles in the literature using the same methodology in a similar population, the results could not be compared with greater precision. In addition, the Youden Index used in the analysis to identify the cutoff point may not be the most appropriate method if the main focus of the study is to maximize true positives (sensitivity) or true negatives (specificity). In the present study, the Youden Index was chosen because it treats sensitivity and specificity with the same weight, based on balance. Likewise, sensitivity analyzes can be considered in different methodologies.

## Conclusion

In conclusion, the cutoff points of 39.125 cm and 39.550 cm for men and 34.725 cm for women for the NC showed good sensitivity and specificity for the diagnosis of MS in the studied population. NC proved to be a simple, easy-to-apply, low-cost and accurate measure for assessing MS in Brazilian rural workers.

## Supporting information

S1 File Minimal data set. (XLSX)

S2 File Analyses. (CSV)
